# Coordination of TGFβ/BMP signaling is associated with the primary cilium

**DOI:** 10.1186/2046-2530-4-S1-P17

**Published:** 2015-07-13

**Authors:** L Lindbæk, CB Warzecha, K Koefoed, JB Mogensen, F Schmid, LB Pedersen, LA Larsen, S Christensen

**Affiliations:** 1 Department of Biology, University of Copenhagen, Copenhagen, Denmark; 2 Department of Cellular and Molecular Medicine, University of Copenhagen, Copenhagen, Denmark

## 

We previously showed that canonical TGFβ signaling is regulated in part by the primary cilium, and that ciliary TGFβ signaling is upregulated in stem cells differentiating into cardiomyocytes [1]. Ciliary signaling was shown to be associated with clathrin-dependent endocytosis at the ciliary pocket for activation of SMAD2/3 transcription factors that associate with and promote SMAD4 translocation to the nucleus for target gene expression. Here we investigated whether other receptor types of the TGFβ/BMP superfamily are associated with the primary cilium and whether ciliary TGFβ/BMP signaling regulates the commitment of stem cells to different lineages. Using retinal pigment epithelium cells, we demonstrate that multiple receptor systems within the TGFβ/BMP superfamily localize to the cilium and the ciliary pocket region, including TGFβ receptors I and II (TGF-RI/II), BMP receptors I and II (BMP-RI/II) as well as two isoforms of Activin II receptors (AcRIIa/b) that can be activated by their corresponding ligands to phosphorylate SMAD2/3, SMAD1/5, ERK1/2, AKT and TAK1 at the ciliary base. Further, knockdown of the feedback inhibitor of SMAD signaling, SMURF1, leads to increased SMAD1/5 phosphorylation at the ciliary base, indicating a major role of the primary cilium in balancing the cellular level of TGFβ/BMP signaling to control cellular processes during development and in tissue homeostasis. Indeed, the level of ciliary TGFβ/BMP signaling was shown to be associated with the ability to commit stem cells to either neurogenesis or cardiomyogenesis, such that downregulation of ciliary signaling promotes neurogenesis and inhibits cardiomyogenesis. Current studies focus on the mechanisms for targeting of TGFβ/BMP superfamily receptors to the primary cilium, trafficking and activation of the receptors within the ciliary compartment, and how these processes contribute to differential cross-talking with other signaling pathways in the cilium and at the pocket region to control cellular processes during development.
